# Large-Scale Wind Disturbances Promote Tree Diversity in a Central Amazon Forest

**DOI:** 10.1371/journal.pone.0103711

**Published:** 2014-08-06

**Authors:** Daniel Magnabosco Marra, Jeffrey Q. Chambers, Niro Higuchi, Susan E. Trumbore, Gabriel H. P. M. Ribeiro, Joaquim dos Santos, Robinson I. Negrón-Juárez, Björn Reu, Christian Wirth

**Affiliations:** 1 Spezielle Botanik und Funktionelle Biodiversität, Universität Leipzig, Leipzig, Deutschland; 2 BGC Prozesse, Max-Planck-Institut für Biogeochemie, Jena, Deutschland; 3 Laboratório de Manejo Florestal, Instituto Nacional de Pesquisas da Amazônia, Manaus, Brazil; 4 Geography Department, University of California, Berkeley, California, United States of America; 5 Climate Sciences Department, Lawrence Berkeley National Laboratory, Berkeley, California, United States of America; 6 Ecology and Evolutionary Biology Department, Tulane University, New Orleans, Louisiana, United States of America; 7 German Centre for Integrative Biodiversity Research (iDiv) Halle-Jena-Leipzig, Leipzig, Deutschland; Lakehead University, Canada

## Abstract

Canopy gaps created by wind-throw events, or blowdowns, create a complex mosaic of forest patches varying in disturbance intensity and recovery in the Central Amazon. Using field and remote sensing data, we investigated the short-term (four-year) effects of large (>2000 m^2^) blowdown gaps created during a single storm event in January 2005 near Manaus, Brazil, to study (i) how forest structure and composition vary with disturbance gradients and (ii) whether tree diversity is promoted by niche differentiation related to wind-throw events at the landscape scale. In the forest area affected by the blowdown, tree mortality ranged from 0 to 70%, and was highest on plateaus and slopes. Less impacted areas in the region affected by the blowdown had overlapping characteristics with a nearby unaffected forest in tree density (583±46 trees ha^−1^) (mean±99% Confidence Interval) and basal area (26.7±2.4 m^2^ ha^−1^). Highly impacted areas had tree density and basal area as low as 120 trees ha^−1^ and 14.9 m^2^ ha^−1^, respectively. In general, these structural measures correlated negatively with an index of tree mortality intensity derived from satellite imagery. Four years after the blowdown event, differences in size-distribution, fraction of resprouters, floristic composition and species diversity still correlated with disturbance measures such as tree mortality and gap size. Our results suggest that the gradients of wind disturbance intensity encompassed in large blowdown gaps (>2000 m^2^) promote tree diversity. Specialists for particular disturbance intensities existed along the entire gradient. The existence of species or genera taking an intermediate position between undisturbed and gap specialists led to a peak of rarefied richness and diversity at intermediate disturbance levels. A diverse set of species differing widely in requirements and recruitment strategies forms the initial post-disturbance cohort, thus lending a high resilience towards wind disturbances at the community level.

## Introduction

Natural disturbances varying in size are suggested as major driver of tree species substitution across space and time, thus influencing vegetation structure, composition and diversity in forest ecosystems [1]–[4]. Disturbance dynamics in neotropical forests are considered to be dominated by small canopy gaps (<2000 m^2^) from small treefall events resulting from small-scale abiotic disturbances [5]–[7] and biotic interactions [8]. In contrast, widespread tree mortality caused by large-scale exogenous disturbances such as forest blowdowns are often regarded as rare events.

However, remote sensing studies confirm a broad range in the size and intensity of wind disturbances representing a continuum without simple distinctions between frequent and episodic events [9]–[11]. In addition, widespread tree mortality associated with blowdowns is more prevalent in the Central and Western Amazon than the Eastern Amazon [12]–[14]. This has revived a classical question [1], [5], [15], [16] on the ecological importance of large canopy gaps (2000 m^2^) and how these influence forest dynamics and tree diversity patterns at the landscape scale [11], [14], [17]–[20].

In Central Amazon forests, there is in fact a large gradient of gap sizes created by blowdowns, which leads to a spatially complex mosaic of successional pathways [10], [20], [21] and gap size distribution varies locally, for example with topography. Studies outside the Amazon report that gap size distributions resulting from wind disturbance vary predictably with elevation [22]–[24], with a greater fraction of large contiguous blowdowns on exposed ridges and plateaus. Blowdowns can raze thousands of trees locally, and with this create a few large gaps (2000 m^2^) as well as many smaller tree-fall gaps (<2000 m^2^) [9], [10], [25].

Large blowdowns and small tree fall gaps may differ substantially in environmental conditions and thus may represent very different starting conditions for tree regeneration. In single-treefall gaps, soil and organic layer disturbance due to uprooting and snapping trees, as well as changes in nutrients and water availability can influence both mortality and recruitment rates as well as other factors vital for the maintenance of tree populations and communities [6], [15], [26]–[28]. Large blowdowns can include a wide range of variability in disturbance severity [22], [29], which may affect the structure and composition in the recovering forest [30]–[33].

As a first order effect, gap formation by adult tree mortality is considered a key process as it increases the local availability of resources, most notably of light [5], [34]. With the resulting increase in high-light microsites [26] forest patches gain the potential to promote light-demanding (pioneers) tree species specialized in colonizing and occupying gaps by efficient dispersal and rapid growth [2], [34]–[36]. Nonetheless, the same traits that convey high growth potential for such light-demanding or pioneer species (e.g. high enzyme activity, low self-shading, and low wood density and construction costs) weaken their competitive strength under undisturbed conditions. Such traits may prevent them from enduring low light conditions, casting shade and outcompeting shade-tolerant and slower-growing (late-successional) trees in the longer term, resulting in relatively short life spans [37]–[41]. Light-demanding species thus depend on frequent gap formation to escape local extinction [2], [5], [6], [34]. However, this binary mosaic view – pioneer versus late-successional species in gaps versus old growth forest – may be too simplistic for understanding the influence of a complex gradient ranging from single tree fall gaps to large blowdowns on landscape-level dynamics of highly-diverse tropical rain forests. Given the complexity of the disturbance mosaic, tropical tree species may not fall into two distinct successional groups but rather form a continuum from pioneer to late-successional strategies [37], [42]–[44]. For example, small gaps (<2000 m^2^) opened up by mortality events of one or a few trees maybe too dark for light-demanding species and colonization may depend on alternative mechanisms, such as growth-release of the understory sapling bank (advanced regeneration), resprouting of damaged trees, and lateral expansion of surviving trees. All three mechanisms may also be relevant in larger gaps (>2000 m^2^) especially given distance-related limitation of seed dispersion. [31], [45]–[48], but here high-light conditions also strongly promote the emergence of pioneer species [33], [49]. However, the threshold disturbance intensity below which colonization by light-demanding species fails is not known. Moreover, it is not known whether there are specialists for intermediate levels of wind disturbance.

By combining a landscape-level approach with a detailed plot-scale analysis of high taxonomic resolution, we analyzed recruitment patterns along a large gradient of disturbance intensity and its potential relevance for maintaining tree species diversity in tropical forests. We hypothesize that the vast species pool of Amazonian forests contains species with strategies intermediate between the extremes of light-demanding and shade-tolerant species, thus enabling the forest vegetation to fully exploit the entire gradient of gap sizes and resource availability. We further hypothesize that a co-occurrence of light-demanding, shade-tolerant and intermediate species leads to a peak in diversity at intermediate disturbance intensities and gap sizes in early regeneration. To address these hypotheses we answered the following questions: *1. Are there predictable patterns of disturbance intensity distributions for large blowdowns, and do these patterns differ between topography classes (valleys, slopes, and plateaus)? 2. Do severe blowdowns exert/promote selective mortality effects at individual and species dimensions? 3. How do pre-blowdown conditions, early vegetation responses and the relative importance of the regeneration mode (establishment from seed, resprouting and growth of survivors) interact with disturbance intensity? 4. What are the implications of these effects on community composition and species coexistence at the landscape scale?*

## Materials and Methods

### Study sites

**Figure 1:**
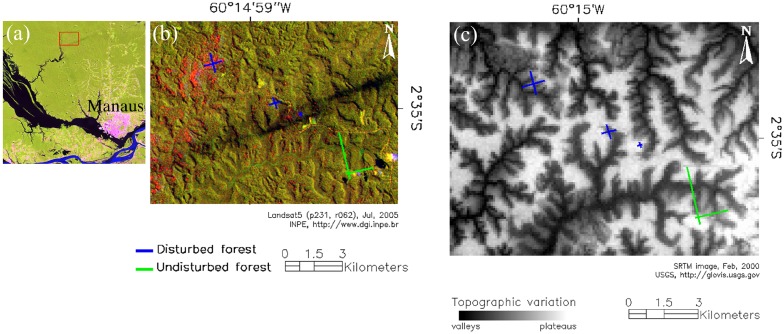
Study areas at the confluence of the Rio Solimões and Rio Negro, Amazonas, Brazil. Legend: (a) Landsat RGB composition of the studied areas (red inset); (b) sampled areas [short-wave infrared reflectance (red channel) indicate the 2005 blowdown tree mortality, measured by quantifying the differences on the no-photosynthetic vegetation (ΔNPV) fraction - SMA analysis]; (c) SRTM elevation model and topographic variation.

We conducted the research at the Estação Experimental de Silvicultura Tropical (EEST) (2°61′S, 60°20′W) of the Instituto Nacional de Pesquisas da Amazônia (INPA) and adjacent-contiguous area (ZF2), which is administered by the Superintendência da Zona Franca de Manaus (SUFRAMA) (2°56′S, 60°26′W), Amazonas, Brazil (Figure 1a). In January of 2005 storms propagating from southwest to northeast of Brazil caused large forest blowdowns across the central Amazon, including ∼2500 ha of forest in the Manaus region [9]. At the ZF2, forest not known to have been previously disturbed near the Rio Cuieiras (a tributary to the Rio Negro) was heavily disturbed, as identified by field surveys and Landsat images (Figure 1).

The entire area is covered by *terra firme* forest. Prior to the 2005 disturbance there was no evidence of human intervention for at least the previous 40 years. In our Landsat chronosequence there was no signal of large natural disturbances affecting the area since 1979. Mean monthly temperature in this region is 26°C with little seasonal variation [50], [51] and annual rainfall is about 2600 mm, with a distinct dry season between July and September [52], [53]. The local topography is undulating with a maximum altitudinal difference of about 140 m (40–180 m a.s.l). Upland plateaus with high clay content (Oxisols) are cut by slopes and valleys dominated by soils with high sand content (Spodosols) and subject to seasonal flooding. The drainage network flows to tributaries of the Rio Negro or directly to the Rio Cuieiras. The *terra-firme* forest is characterized by a closed canopy with high tree species diversity [54]–[57] and a dense understory with abundant acaulescent palm species in plateaus and canopy palm species in valleys [57], [58]. In this region, forest compositional and structural variations are correlated with water stress, soil and topography [54], [59]–[61].

### Forest inventory

We sampled three different patches directly affected by the 2005 blowdown (hereafter referred to as disturbed forest). To assess the entire disturbance gradient we installed three pairs of transects, measuring 200, 600 and 1000 m length by 10 m width respectively, for a total of six transects (Figure 1b – blue lines). We established 144 sub-plots of 10×25 m (total of 3.6 ha) within the 6 transects. At each sub-plot, we determined several measures of forest structure for trees with diameter at breast height (DBH) ≥10 cm. Diameters of live uprooted and damaged-snapped trees were usually measured above the DBH (1.3 m height) to avoid lesions and mechanical obstructions, such as trunks, branches, soil, etc. For species with buttressed and aerial roots, we measure the diameter just above these obstructions.

Considering previous studies [26], [46], [62] and field observations, we identified resprouting stimulated by mechanical injuries in individuals following uprooting and snapping (partial or total rupture of the crown). For botanical identification we collected samples from at least one individual of each species and for subsequent re-measurement we tagged all trees. We carried out identification to the species level [63]–[65] when possible, by comparing the collected material with specimens at the INPA and IFAM (Instituto Federal de Educação, Ciência e Tecnologia do Amazonas) herbariums, and also with the EEST botanical collection. Reproductive material (flowers and/or fruits) was collected when available, and added to the IFAM herbarium and to the EEST botanical collection. Our research did not involve endangered or protected species and no special permits were required to access the ZF2. We carried the forest inventory between June and September of 2009. Botanical samples were collected in two campaigns, in 2009 and 2011.

We used measurements from an undisturbed contiguous area (hereafter referred to as undisturbed forest) to compare with our data for the disturbed forest (Figure 1b – green lines). In this area, two transect plots measuring 20×2.500 m (10 ha) were installed in 1996 [66] and forest structure and dynamics are monitored in 250 sub-plots (20×20 m) using measures of growth, recruitment and mortality made every two years since 2000 [52], [67]. We used data from 196 (total 7.8 ha) sub-plots in an area not affected by clouds in both 2004 and 2005 Landsat images, and estimated structural parameters at these sites in 2008. We used census data from these sites for the years 2004–2006 to obtain background forest mortality rates for an area not directly affected by the 2005 blowdown (as confirmed using our Landsat ΔNPV maps) [10]. Although much of the Amazon experienced drought in 2005 with potential effects on mortality [68], [69], the region we studied was not unusually dry during this period [9], [53].

The transects established in disturbed and undisturbed forests were perpendicularly oriented in north-south and east-west directions (Figure 1b). These transects cross several toposequences in order to represent the floristic and structural variation between plateaus, slopes and valleys (Figure 1c). For classifying sub-plots into topographic classes we used field observations, apparent soil texture, slope and altitude [56], [57], [66]. Slope was measured with a clinometer and sub-plot altitude data was assessed by using a Global Positioning System (GPS) receiver (*Trimble Nomad 900*) and a digital elevation model (Shuttle Radar Topographic Mission -SRTM) (http://glovis.usgs.gov). Higher altitude areas (>80 m a.s.l.) with lower slope (∼0–10°) and higher clay content soils were classified as plateaus. Areas with slope >10°, mixed sand/clay texture soils, and located in interfluve areas were classified as slopes. Areas with low elevation and relief with sandy soil and direct contact with perennial or intermittent water streams were classified as valleys (Figure 1). In the Manaus region, valleys can be also characterized by the high abundance of some palm species (e.g. *Mauritiella aculeata* (Kunth) Burret, *Oenocarpus bataua* Mart. and *Manicaria saccifera* Gaertner) and for being partially inundated during the rainy season. In the disturbed forest we classified 44 (1.1 ha), 73 (1.8 ha) and 27 (0.7 ha) sub-plots in plateaus, slopes and valleys, while in the undisturbed forest plateaus, slopes and valleys represented 49 (2.0 ha), 96 (3.8 ha) and 51 (2.0 ha) sub-plots, respectively.

In both disturbed and undisturbed forests we counted dead trees and measured their DBH. In the disturbed forest, we classified mode of tree death related to the blowdown as snapped, uprooted or standing dead according to previous studies [9], [25], [62]. For both disturbed and undisturbed forests, we estimated tree density (trees ha^−1^) and basal area (m^2^ ha^−1^). For the disturbed forest, we additionally estimated mean DBH (cm) and wood density (g cm^3^) for each sub-plot. We compiled species wood density from available literature [70]–[73] to check for possible correlations between disturbance intensity measures and wood density variation. For species for which data were not available or the identification was only possible at the genus level, we used genus-level obtained by calculating the mean wood density of local congeneric species. For six trees (snapped or without leaves), we used family-level obtained by calculating the mean wood density of the recorded species from the respective family.

### Satellite data and disturbance parameters

We used Landsat-5 thematic mapper images (Path 231, Row 062), obtained from Brazil’s National Institute for Space Research (INPE, http://www.dgi.inpe.br/CDSR/) and the United States Geological Survey (USGS, http://glovis.usgs.gov) to assess disturbance intensity over the disturbed area, directly affected by the 2005 blowdowns. We first georeferenced (400 control points per image) the images using the NASA Geocover data set as a basemap (https:/zulu.ssc.nasa.gov/mrsid/) and several reference points across the EEST route were collected using a GPS (*Trimble Nomad 900*); we applied a mask to remove clouds, cloud shadows, land use, and water bodies; and removed smoke and haze contamination with the Carlotto technique [74]. Further, we carried out a spectral mixture analysis (SMA) [75] to determine the fractions of green vegetation (GV), nonphotosynthetic vegetation (NPV) and shade. We normalized the pixels without shade as GV/(GV + NPV) and NPV/(GV + NPV), resulting in shade-normalized GV and NPV fractions [76]. The ΔNPV reflects changes in nonphotosynthetic vegetation (NPV, wood, dead vegetation and surface litter) before and after the storm and was calculated as NPV2005-NPV2004. We carried out SMA analysis with the Environment for Visualizing Images software (ENVI, ITT Industries, Inc, Boulder CO, USA).

We assessed disturbance intensity in the disturbed forest by estimating three disturbance parameters on a per-pixel basis (30×30 m): sub-plot mortality, mortality in the area surrounding the sub-plot (hereafter referred to as neighboring mortality) and gap size; these three disturbance parameters derived from tree mortality measures (in percentage) obtained from a mortality estimation model which has the ΔNPV as predictor [Y = 103.22*ΔNPV-3.32] (*r^2^* = 0.8 and *P*<0.001) [9]. We used the four corner coordinates (northeast, northwest, southeast and southwest) to rasterize polygons representing our sub-plots on the ΔNPV image. The sub-plot’s ΔNPV was obtained by calculating the area weighted mean ΔNPV value of those pixels included in its respective rasterized polygon. To increase the accuracy of this estimation, we resampled the ΔNPV image from its native resolution of 30×30 m to a 3×3 m grid size, which allowed improved estimation of the relative area within each pixel contributing to the total sub-plot area.

We applied the sub-plot’s weighted mean ΔNPV in our mortality estimation model [9] to assess tree mortality at sub-plot level in the two forests. For the disturbed forest we calculated the neighboring mortality with the same equation by applying the mean ΔNPV value of the eight pixels directly adjacent to that pixel which included the geographic coordinates of the sub-plot’s central point. Additionally, we estimated gap size as the area of virtual polygons formed by groups of continguous pixels with ΔNPV≥0.16. In these forests a ΔNPV≥0.16 corresponds to a mortality rate of ∼13% (∼6 trees/pixel), which is conservatively above the minimum rate observed from smaller treefall gaps in local old growth forests (∼2%) [67], [77], and above the single-pixel mortality gaps obtained from field-measured mortality (∼5%) [10], [25]. In our mortality model [Y = 103.22*ΔNPV-3.32], ΔNPV<0.032 produced negative mortality estimates, which occurred exclusively in the undisturbed forest and valleys of the disturbed forest. Because the model was adjusted only for disturbed areas, negative ΔNPV values cannot be interpreted as real gain in PV (or forest regrowth). In order to avoid negative mortality estimates, we applied a threshold (ΔNPV<0.032) below which we assumed mortality was zero for sub-plots. Neighboring mortality and gap size were only estimated for the disturbed forest. We computed sub-plot and neighboring mortality by using tools from the *raster* [78] and *maptools* [79] packages implemented in the R (*version 3.0.1*) software platform [80]. We computed gap size with the SAGA software (*version 2.0.8* http://www.saga-gis.org).

### Statistical analysis

#### Disturbance patterns

To check for recognizable relationships between disturbance patterns and topography, we compared structural (tree density and basal area) and disturbance intensity (sub-plot mortality) of the different forests. For the disturbed forest, we tested for the effects of disturbance intensity measures (sub-plot mortality, neighboring mortality and gap size) on subsequent structural and floristic variation in the different topographic classes of the disturbed forest.

To assess structural differences among topographic classes within the two forests, we classified sub-plots from the disturbed forest in two levels of disturbance. Non- and low-disturbance sub-plots (ΔNPV<0.16; hereafter referred as low disturbance) [10] were compared with sub-plots that experienced disturbance above this threshold (ΔNPV>0.16; hereafter referred to as high disturbance). We compared ΔNPV and structural measures of the two forests with factorial ANOVA. We used a Post-hoc Tukey test to evaluate significant differences among topographic classes. In disturbed forest, we related mode of death (snapped, uprooted and standing dead) among topographic classes with linear regressions and assessed whether mode of death is still recognizable and related to sub-plot mortality. With one-way ANOVA, we compared the mean DBH of dead trees among modes of death. Additionally, we tested for the effect of the disturbance intensity on subsequent structural variation by relating tree density and basal area to sub-plot mortality with linear regressions. The same analysis was applied to test for possible correlations between mean DBH of recorded dead trees and sub-plot mortality.

#### Selective mortality patterns

To address our questions related to selective mortality effects at individual and species level, we compared the size distribution of trees from the disturbed with the undisturbed forest. For the disturbed forest, we described the variation of genera’s importance at low and high disturbance levels, and related species diversity measures to sub-plot mortality.

Due to the lower disturbance intensity that has been observed in valleys, and also to pre-blowdown distinct structural and floristic characteristics of the vegetation in the valleys, we only considered sub-plots established on plateaus and slopes for these analyses. To assess the influence of disturbance intensity on size distribution of trees and on the importance of genera, we also considered the classification described in section 2.4.1. We assessed the disturbance intensity effects on the size distribution of trees using Chi-squared tests comparing the diameter distribution of live and dead trees within the disturbed and undisturbed forests, and also within the levels of disturbance described above. For the disturbed forest, we additionally related the mean DBH of live trees to sub-plot mortality. We described community demographic patterns at the genus level for low and high disturbance sub-plots by estimating the Importance Value Index (IVI), calculated as the sum of relative density, frequency and dominance (basal area) of congeneric species [81]. To assess the effects of disturbance intensity on diversity patterns, we related with linear and polynomial regressions, species richness, Shannon diversity and species rarefaction to sub-plot mortality. These three measures were calculated for groups of five sub-plots, ranked and grouped by their sub-plot mortality value (23 in total). With this we avoided the tree density effect observed in those sub-plots with high mortality values, and thus few trees (e.g. with three trees); and were able to look for patterns at the community level.

#### Disturbance gradient effects and vegetation responses

To address our last two questions, on vegetation responses along the disturbance gradient and changes on community composition, we assessed the importance of resprouters and fast growing or pioneer species, and checked for variations in species composition with disturbance intensity measures.

With linear regressions we assessed the effect of sub-plot mortality, neighboring mortality and gap size on the fraction of resprouters; and effect of sub-plot mortality on mean wood density. We tested with one-way ANOVA for wood density and size differences between resprouters and non-resprouters. Further, we related the fraction of pioneer species basal area to sub-plot mortality and mean wood density; and related the fraction of pioneer species with neighboring mortality and gap size. We consider as pioneers, light-demanding/fast-growing species typical for the region from the genera *Cecropia*, *Conceveiba*, *Inga*, *Laetia*, *Miconia*, *Pourouma*, *Tachigali* and *Vismia*. To reduce the influence of possible pioneer trees that were established before the blowdown, we only accounted for trees in these genera with DBH≤25 cm.

To test for the effect of disturbance intensity on floristic composition, we used nonmetric multidimensional scaling (NMDS) to reduce the dimensionality of the community species composition [82], [83]. Ordinations were computed with two axes from one dissimilarity matrix generated by the abundance of all recorded species. We used Mantel-tests (*P*<0.001) to test for the significance of the NMDS analysis. Finally, sub-plot mortality, neighboring mortality and gap size were used as predictors in linear regressions to assess the effects of disturbance intensity measures on the floristic composition variation detected by the first NMDS axis. Finally, to look in detail at possible variations on genera composition driven by the disturbance intensity, we fit a cubic smoothing spline function (degrees of freedom = 3) relating the abundance of the 25 most important genera (based on IVI values) to sub-plot mortality. As in our previous analyses (see section 2.4.2) only sub-plots established on plateaus and slopes were used for these last two analyses. All statistical analyses were performed in R (*version 3.0.1*) software platform [80]. We used the vegan package [84] for estimating diversity indices, species curves and to process the NMDS analysis. All other tests were custom written.

## Results

### Disturbance patterns

In the disturbed forest we sampled 1944 live and 363 dead trees. Our structural measures confirm a correlation between ΔNPV and tree mortality, and indicate that vegetation damage was partially controlled by topography.

Tree density and basal area in the sub-plots (250 m^2^) of the disturbed forest ranged from 3 to 33 trees and from 0.08 to 2.23 m^2^, respectively. Mean tree density and basal area in the undisturbed forest was higher than in the low and high disturbance sub-plots (Table 1). Mean tree density and basal area in slopes from the high disturbance was lower than in slopes from the low disturbance and undisturbed forest (Table 1).

**Table 1:** Summary statistics (mean±99% CI) of structure and disturbance intensity measures for different levels of wind-disturbance in a forest disturbed by a large blowdown, and an undisturbed forest, in Amazonas, Brazil.

	TC	NS	TD	BA	ΔNPV	SP	NG	GS
UN	All	196	593±28*	27.7±2.1*	0.038±0.016*	3.7±0.8		
	Plateaus	49	632±46	29.1±4.4	0.048±0.036	4.6±1.9		
	Slopes	96	590±34	26.8±2.9	0.047±0.022	4.2±1.3		
	Valleys	51	560±44	28.2±3.7	0.011±0.030	2.0±0.9		
LD	All	75	583±46*	26.7±2.4*	0.050±0.016*	4.4±0.9	5.4±1.4*	1.1±0.9*
	Plateaus	12	643±5	28.0±0.2	0.048±0.002	5.2±0.1	8.4±0.2	0.4±0.04
	Slopes	39	615±99	27.1±5.1	0.054±0.033	4.8±1.9	5.9±2.8	0.6±1.5
	Valleys	24	500±78	25.3±5.3	0.046±0.029	3.2±2.0	3.2±2.5	1.9±3.4
HD	All	69	462±62*	19.9±4.9*	0.312±0.040*	28.9±4.2*	25.0±4.2*	12.6±2.7*
	Plateaus	32	554±98	25.5±8.9	0.326±0.064¥	30.4±6.7¥	26.0±5.3¥	9.2±3.3¥
	Slopes	34	426±76¥	14.5±4.2¥	0.307±0.054¥	28.5±5.6¥	24.7±6.8¥	16.3±3.9¥
	Valleys	3	600±158	21.5±7.0	0.208±0.040¥	18.2±4.1	16.8±7.8	6.6±13.3

Legend: UN- undisturbed forest; LD- low disturbance (up to 13% of mortality); HD- high/severe disturbance (greater than 13% of mortality); TC- topographic classes; NS- number of sampled sub-plots; TD- tree density (trees ha^−1^); BA- basal area (m^2^ ha^−1^); SP- sub-plot mortality (%); NG- neighboring mortality (%); GS- gap size (ha).

*Significant differences (ANOVA) among levels of disturbance (*P*<0.001).

¥ Significant differences (Tukey HSD) between same topographic classes (*P*<0.001).

In the disturbed forest, ΔNPV ranged from −0.213 to 0.696 with an overall mean value of 0.176±0.037 (mean±99% CI), higher than from the undisturbed forest (0.038±0.016; ranging from −0.168 to 0.202). Estimated sub-plot mortality in the disturbed forest ranged from 0 to 69.9% with an overall mean value of 16.2±3.4%, higher than from the undisturbed forest (3.7±0.8%; ranging from 0 to 30.5). In the undisturbed forest, mortality rate computed from field data (2004–2006) was 1.6±0.7% year^−1^ and correlated positively but weakly with the estimated sub-plot mortality (*r^2^* = 0.04; *P* = 0.004; Pearson’s *r* = 0.20). Plateaus, slopes and valleys from high disturbance sub-plots had higher mean ΔNPV and sub-plot mortality in comparison to low disturbance and undisturbed forest (Table 1). Plateaus and slopes from the high disturbance also had higher neighboring mortality and gap size than plateaus and slopes from the low disturbance. The disturbed sub-plots were set in gaps that ranged in size from 0–22.6 ha (6.5±2.3), which correlated positively with mean sub-plot mortality (*r^2^* = 0.37; *P*<0.001; Pearson’s *r* = 0.60). During the analyzed period we did not find gaps larger than three pixels (2700 m^2^) in the undisturbed forest.

In the disturbed forest the density of dead trees ranged from 0 to 360 trees ha^−1^ and the mean (101±22 trees ha^−1^) varied among topographic classes (*F* = 8.74; *P*<0.001). The mean density of dead trees in plateaus (104±43 trees ha^−1^) was similar to that for slopes (120±31 trees ha^−1^) (*P* = 0.536), but exceeded the mean density observed in valleys (44±31 trees ha^−1^) (*P*<0.01, both). The total number of dead trees, both snapped and uprooted, correlated positively with sub-plot mortality in slopes in plateaus. In the disturbed forest plateaus and slopes, the density of live trees and basal area were negatively correlated with sub-plot mortality, while valleys were not affected (Table 2).

**Table 2:** Linear regressions and Pearson’s correlation relating structural variables to mortality intensity in a forest disturbed by a large blowdown, in Amazonas, Brazil.

	Structure variables	Topographic classes¥	N. of trees	Regression parameters*					
				a	b	F	*r* ^2^	*P*	Pearson’s *r*
Dead trees	N. of dead trees	Plateau	114	1.5191	0.0456	6.00	0.125	0.018	0.354
		Slope	219	2.1073	0.0564	13.5	0.159	<0.001	0.4
		Valley	30	0.8224	0.059	2.43	0.052	0.131	0.297
	N. of snapped trees	Plateau	51	0.2713	0.0378	9.17	0.18	0.004	0.423
		Slope	92	1.055	0.0129	2.08	0.028	0.153	0.168
		Valley	19	0.5055	0.0405	2.38	0.086	0.135	0.294
	N. of uprooted trees	Plateau	44	0.8259	0.0074	0.34	0.008	0.559	0.09
		Slope	105	0.5709	0.0548	16.45	0.188	<0.001	0.434
		Valley	7	0.138	0.0248	0.86	0.033	0.362	0.182
	N. of standing dead trees	Plateau	19	0.4218	0.0004	<0.01	−0.023	0.954	0.008
		Slope	22	0.4813	−0.0113	5.48	0.071	0.022	−0.267
		Valley	4						
Live trees	Tree density (trees ha^−1^)	Plateau	637	648.69	−3.000	2.46	0.055	0.123	−0.235
		Slope	962	658.28	−8.290	29.36	0.293	<0.001	−0.54
		Valley	345	494.97	3.3030	0.47	0.018	0.5	0.135
	Basal area (m^2^ ha^−1^)	Plateau		31.57	−0.2286	2.23	0.051	0.143	−0.224
		Slope		29.14	−0.4966	36.2	0.337	<0.001	−0.582
		Valley		25.25	−0.0742	0.05	0.002	0.819	−0.046

*Model: y = a+bx+Єi.

¥ Degrees of freedom for the topographic classes: plateaus (44); slopes (73); valleys (27).

### Selective mortality patterns

Our data show that the blowdowns promoted subsequent changes in forest structure, genera substitution and species demography. Four years after the blowdown event, differences in size-distribution, genera importance and diversity measures still correlated with levels of disturbance.

In the disturbed forest, the DBH of recorded dead trees ranged from 10 to 120 cm, with mean value (27.5±2.3 cm) higher than from that from live trees of both disturbed (20.6±2.3 cm) and undisturbed forest (21±0.7 cm). DBH of dead trees did not vary among modes of death (ANOVA *F* = 2.37; *P* = 0.125), but for snapped and uprooted trees had a low correlation with sub-plot mortality (*r^2^* = 0.02; *P* = 0.029; Pearson’s *r* = 0.13), with a non-variable mean value among modes of death (ANOVA *F* = 2.37; *P* = 0.125).

**Figure 2:**
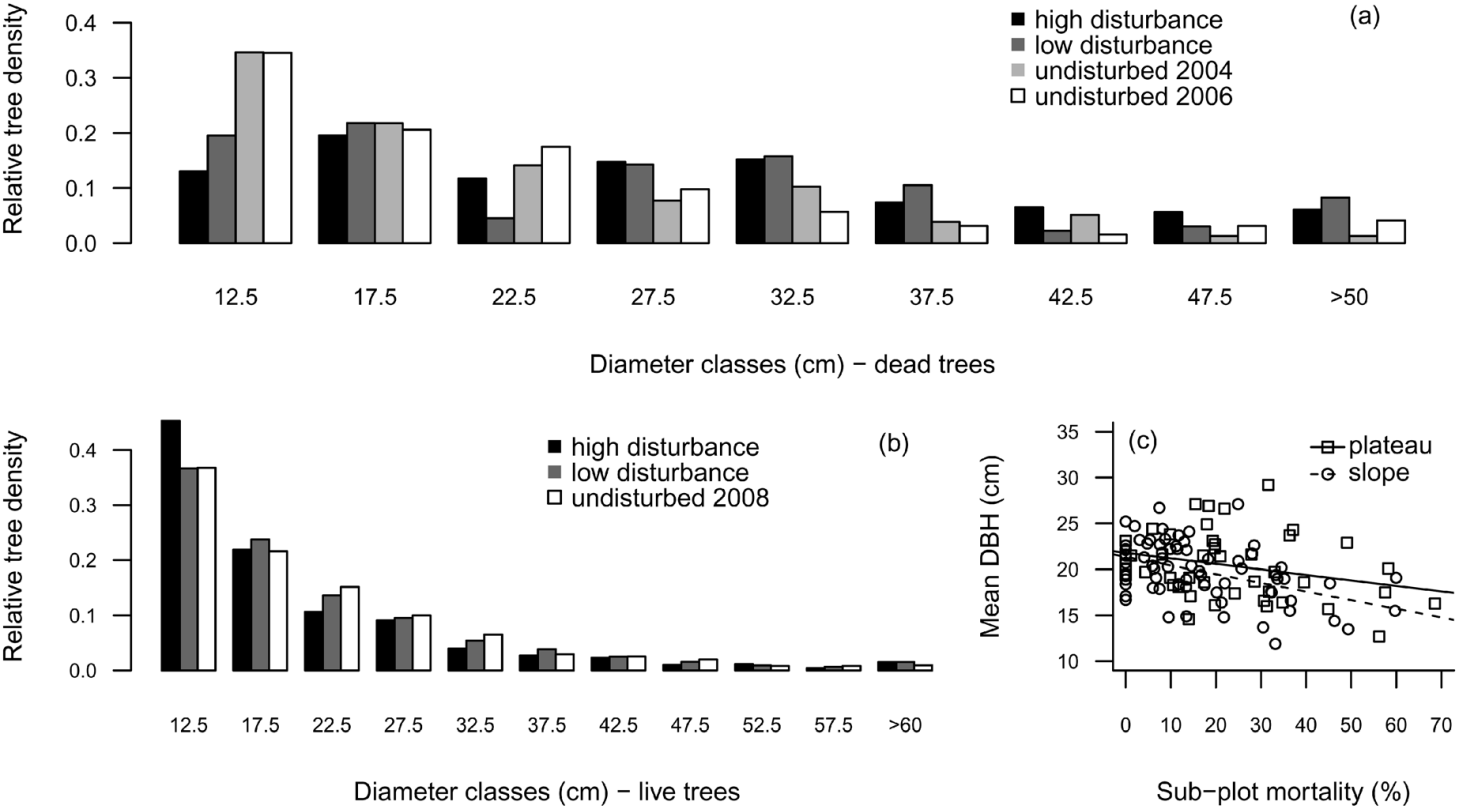
Tree size distribution (DBH≥10 cm) in plateaus and slopes of a forest disturbed by a large blowdown and an undisturbed forest, in Amazonas, Brazil. Legend: (a) diameter distribution of dead trees; (b) diameter distribution of live trees; (c) mean DBH of live trees in disturbed plateaus (*r* ^2^ = 0.05; *P* = 0.06; Pearson’s *r* = −0.277) and slopes (*r* ^2^ = 0.19; *P<*0.001; Pearson’s *r* = −0.451) related to sub-plot mortality.

Even four years after the blowdown event, the DBH distribution comparisons indicated size distribution differences between trees of the disturbed and the undisturbed forests. The DBH distribution of dead trees in both low- and high-disturbance sub-plots (Figure 2a) did not follow that of the undisturbed forest before (2004) and after (2006) the blowdown event (Chi-squared test, *P*<0.001). While the undisturbed forest had a higher abundance of dead trees within the smaller diameter classes, low and high disturbance forests had higher abundance of large trees exhibiting a log-normal distribution. The DBH distribution under low and high disturbance was also different (Chi-squared test, *P = *0.017). Within live trees, both disturbed and undisturbed forests had higher abundance of small trees and exhibit the typical negative exponential DBH distribution of tropical forests (Figure 2b). The DBH distribution of live trees under low disturbance was similar to that of the undisturbed forest (*P = *0.490), while under high disturbance forest it diverged from that observed within trees from the undisturbed and low disturbance forest (*P*<0.001). The overall mean DBH of live trees was negatively related to sub-plot mortality in plateaus and slopes of the disturbed forest (Figure 2c).

**Figure 3:**
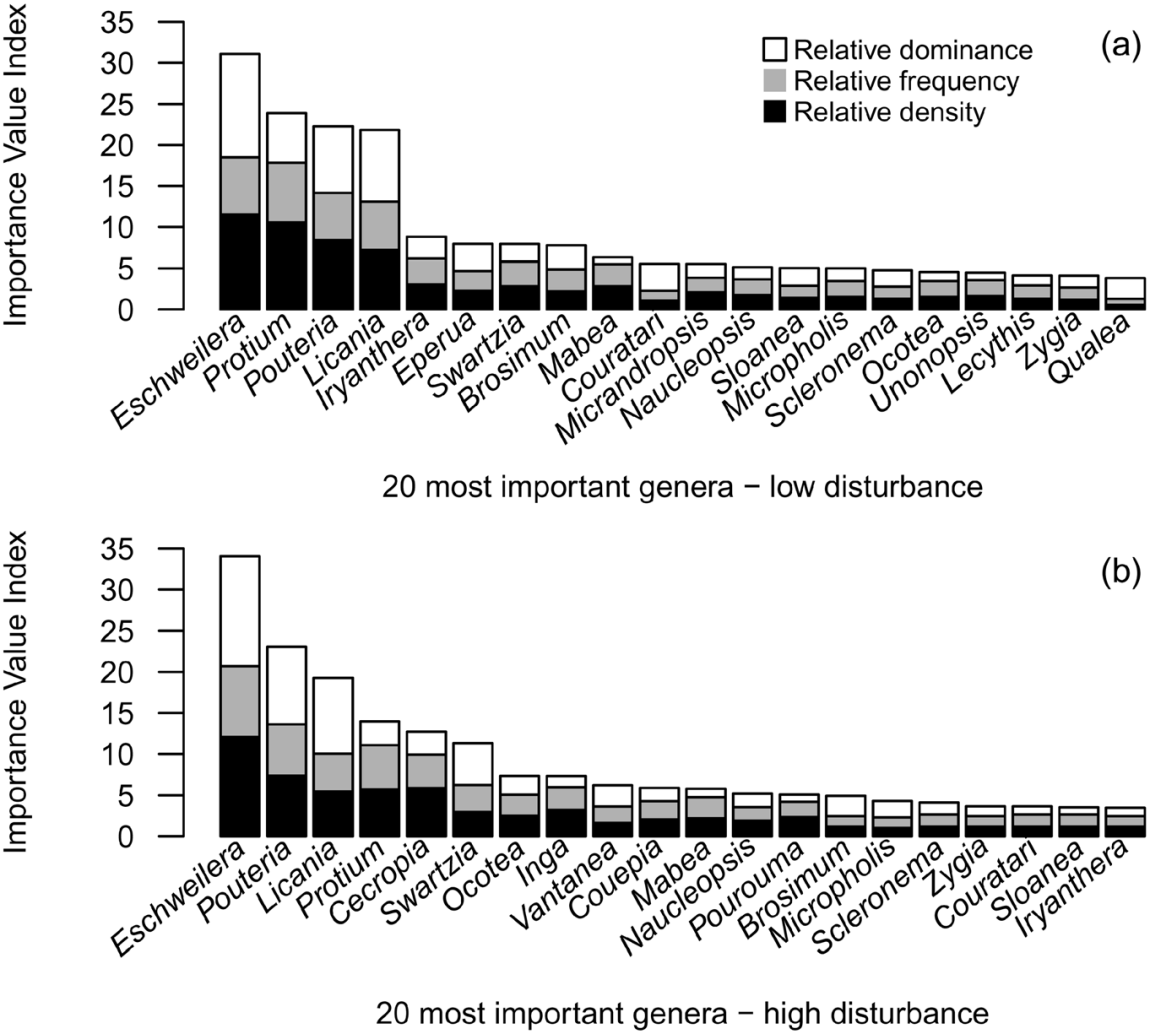
Genera importance ranking over plateaus and slopes of a forest disturbed by a large blowdown, in Amazonas, Brazil. Legend: (a) Importance Value Index (IVI) of the 20 most important genera recorded under low disturbance (up to 13% of tree mortality); (b) IVI of the 20 most important genera recorded under high disturbance (up to 70% of tree mortality).

In the disturbed forest, recorded trees were distributed in 51 families, 158 genera and 324 species or morphotypes. A total of 54 species (∼16%) just occurred on plateaus, while 55 (∼17%) and 20 (∼6%) were restricted to slopes and valleys, respectively. Fifty eight species (∼18%) occurred in all topographic classes. Fabaceae (66 species), Sapotaceae (26) and Lecythidaceae (23) were the richer families in number of species and together summed ∼40% of all recorded trees and ∼36% of the total richness. Eighteen families were represented by just one species. 129 species were represented by only a single tree. Heavily damaged sub-plots, especially those in gaps with large amounts of necromass, often had high density of lianas. Field observations showed that gaps in which wood decay was more advanced generally had a dense understory with seedlings dominated by the genera *Casearia* and *Laetia* (Salicaceae), *Cecropia* and *Pourouma* (Urticaceae), *Conceveiba* and *Croton* (Euphorbiaceae), *Inga* and *Tachigali* (Fabaceae), *Miconia* (Melastomataceae), *Tapirira* (Anacardiaceae) and *Vismia* (Hypericaceae). Genera demographic analyses revealed differences between low (total of 258 species) and high disturbance (215). Ten genera among the 20 most important genera from the high and the low disturbance levels were different (Figure 3). The high disturbance level had both typical light-demanding/fast-growing (e.g. *Cecropia*, *Inga* and *Pourouma*) and shade-tolerant/slower-growing (e.g. *Brosimum*, *Pouteria*, *Sloanea* and *Vantanea*) exclusive genera within the 20 most important genera. *Eschweilera*, *Licania*, *Pouteria* and *Protium* had the highest IVI in both disturbance levels, but the contributions of the IVI estimators of these and other common genera varied between disturbance levels.

**Figure 4:**
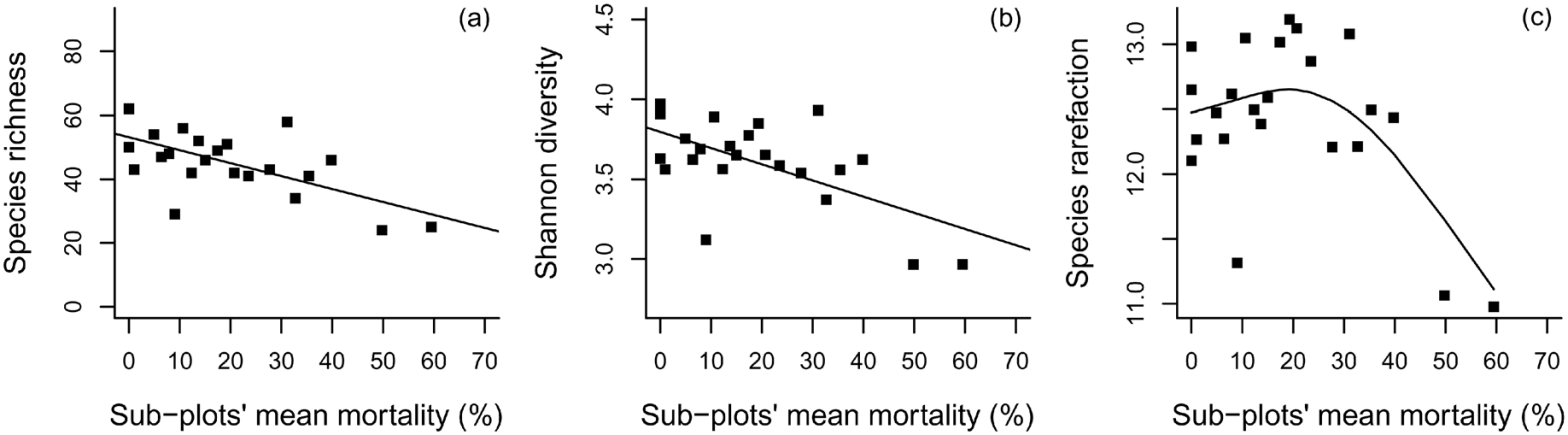
Species richness and diversity measures related to a mortality gradient in plateaus and slopes of a forest disturbed by a large blowdown, in Amazonas, Brazil. Legend: (a) species richness; (b) Shannon diversity; (c) species rarefaction.

Species richness per sub-sub-plot varied from 3 to 25 species in the disturbed forest. For groups of plots, species richness was negatively related to sub-plot mortality (*r^2^* = 0.39; *P*<0.001; Pearson’s *r* = −0.64) (Figure 4a). Shannon diversity also correlated negatively with sub-plot mortality (*r^2^* = 0.39; *P* = 0.002; Pearson’s *r* = −0.61). Nonetheless, at intermediate disturbance (20–50% of tree mortality), Shannon diversity was slightly lower or even similar to that from the less- and non-disturbed sub-plots (Figure 4b). Species rarefaction curve along the mortality gradient indicated that intermediate-disturbance areas can be more diverse than both heavily and undisturbed areas (Figure 4c).

### Disturbance gradient effects and vegetation responses

Of the live trees recorded in the disturbed forest, 206 (∼11% of total sampled) had crown or trunk injuries and 191 (∼10%) had one of the mechanisms indicative of resprouting. 151 trees (∼7%) belonged to light-demanding/fast-growing pioneer genera, and the mortality gradient appeared to amplify forest niches that lead to changes in species composition.

**Figure 5:**
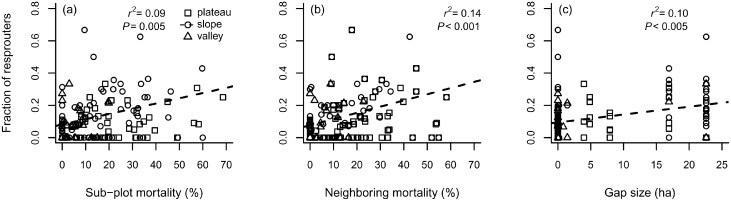
Fraction of resprouting tree species related to disturbance intensity measures from a forest disturbed by a large blowdown, in Amazonas, Brazil. Legend: (a) fraction of resprouters related to sub-plot mortality (slopes Pearson’s *r = *0.33); (b) fraction of resprouters related to neighboring mortality (slopes Pearson’s *r = *0.38); (c) fraction of resprouters related to gap size (slopes Pearson’s *r = *0.33).

Resprouting trees were recorded in all the topographic classes and within different species. In slopes, the fraction of resprouters related positively to sub-plot mortality as well as to other disturbance intensity measures (Figure 5). In plateaus and slopes, the mean wood density correlated negatively with sub-plot mortality (*r^2^* = 0.03; *P*<0.035; Pearson’s *r* = *−*0.19), but resprouters and non-resprouters had no significant differences in wood density (*F* = 0.262, *P* = 0.609), but did have in DBH (*F* = 4.87, *P* = 0.027).

**Figure 6:**
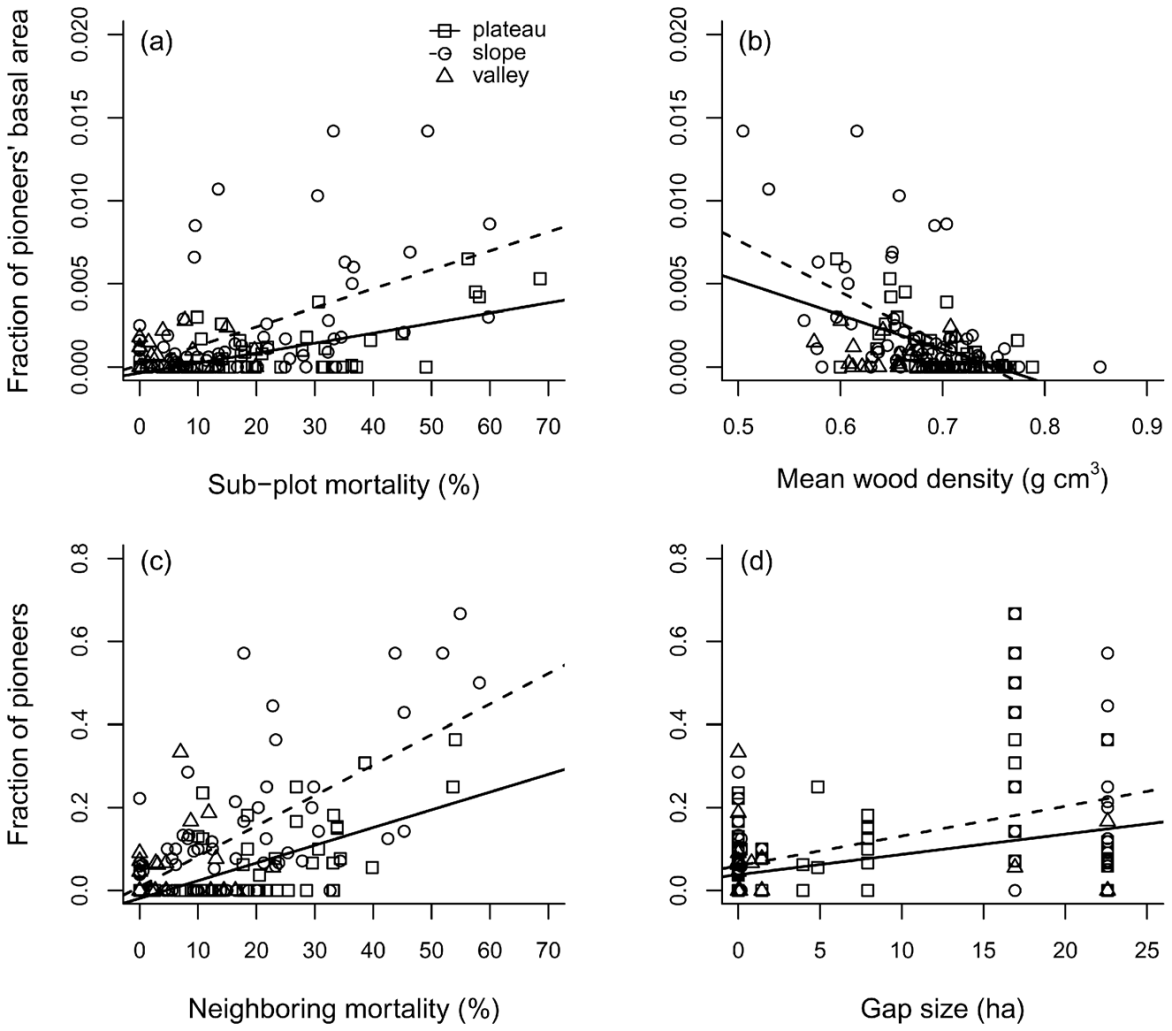
Pioneer (light-demanding and fast-growing) tree species importance related to disturbance intensity measures and wood density in a forest disturbed by a large blowdown, in Amazonas, Brazil. Legend: (a) fraction of pioneers’ basal area related to sub-plot mortality; (b) fraction of pioneers’ basal area related to sub-plot mean wood density; (c) fraction of pioneers related to neighboring mortality; (d) fraction of pioneers related to gap size.

In general, light-demanding/fast-growing genera (e.g. *Cecropia*, *Conceveiba*, *Inga*, *Casearia*, *Miconia*, *Pourouma*, *Tachigali*, *Tapirira* and *Vismia*) contributed less to total basal area in less disturbed sub-plots, while their basal area was positively related to sub-plot mortality in plateaus (*r^2^* = 0.39; *P*<0.001; Pearson’s *r = *0.63) and slopes (*r^2^* = 0.27; *P*<0.001; Pearson’s *r = *0.52) (Figure 6a). In contrast, the fraction of basal area contributed by these fast-growing species was negatively related to sub-plot mean wood density, also exclusively in plateaus (*r^2^* = 0.30; *P*<0.001; Pearson’s *r* = *−*0.56) and slopes (*r^2^* = 0.31; *P*<0.001; Pearson’s *r* = *−*0.57) (Figure 6b). The fraction of these typical pioneer species was positively related to neighboring mortality in plateaus (*r^2^* = 0.32; *P*<0.001; Pearson’s *r = *0.58) and slopes (*r^2^* = 0.46; *P*<0.001; Pearson’s *r = *0.67) (Figure 6c). Gap size was also positively related to these group of species in plateaus (*r^2^* = 0.14; *P = *0.013; Pearson’s *r = *0.34) and slopes (*r^2^* = 0.21; *P*<0.001; Pearson’s *r = *0.44) (Figure 6d).

**Figure 7:**
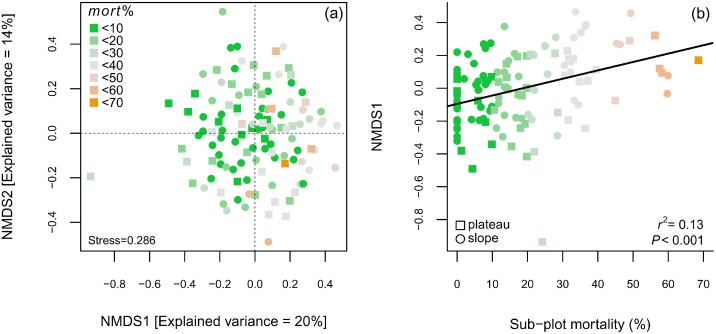
NMDS ordination diagram of 117 sub-plots sampled in plateaus and slopes of a forest disturbed by a large blowdown, in Amazonas, Brazil. Sub-plot mortality caused by the blowdown is highlighted by the color-scale. Legend: (a) NMDS scores computed from a dissimilarity matrix with the abundance of all recorded species; (b) first NMDS axis related to sub-plot mortality.

The NMDS stress value was 0.286, and the first two axes captured 34% of the floristic variation (Figure 7a and Table 3). Floristic similarities within heavily damaged sub-plots increased due to the higher abundance of light-demanding and fast-growing species. Sub-plot mortality related positively to the variations in species composition captured by the first NMDS axis (Figure 7b). The same pattern was observed for neighboring mortality and gap size, which correlated more strongly (Table 3).

**Table 3:** NMDS and linear regression relating measures of disturbance intensity to the floristic composition of a forest disturbed by a large blowdown, in Amazonas, Brazil.

NMDS	Mantel-test*		NMDS [Axis1] related to disturbance measures¥				
Stress	Axis 1	Axis 2	Predictors	F	*r^2^*	*P*	Pearson’s *r*
0.286	0.442	0.367	SP	18.15	0.129	<0.001	0.370
			NG	21.81	0.159	<0.001	0.400
			GS	25.56	0.175	<0.001	0.427

Legend: SP-sub-plot mortality; NG-neighboring mortality; GS-gap size.

*Related to species distance matrix and NMDS axis scores.

¥ Linear regressions with NMDS (Axis1) as dependent variable and disturbance intensity measures as predictors.

**Figure 8:**
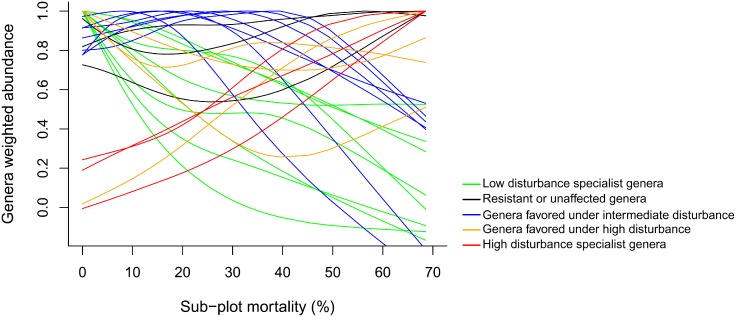
Genera abundance related to a mortality gradient in plateaus and slopes of a forest disturbed by a large blowdown, in Amazonas, Brazil. Legend: low disturbance specialist genera- *Brosimum*, *Eperua*, *Iryanthera*, *Micrandropsis*, *Micropholis*, *Pouteria*, *Protium* and *Unonopsis*; resistant or unaffected genera- *Couratari*, *Ocotea* and *Sloanea*; genera favored under intermediate disturbance- *Eschweilera*, *Lecythis*, *Licania*, *Naucleopsis*, *Swartzia*, *Qualea* and *Zygia*; genera favored under high disturbance- *Couepia*, *Mabea*, *Scleronema* and *Vantanea*; high disturbance specialist genera- *Cecropia*, *Inga* and *Pourouma*.

The demographic patterns of the 25 most important genera in the community (identified in Figure 3) and the variations in species composition (Figure 7) corroborate our results related to genera abundance variation in respect to disturbance intensity (Figure 8). The genera abundance curves show that these 25 genera are not equally distributed along the landscape and that part of this variation was regulated by variations in sub-plot mortality. Interestingly, the abundance of these genera varied along the mortality gradient revealing five distinct groups (identified using colors in Figure 8) which had their optimum under specific disturbance intensities. This pattern indicates that there exist specialist guilds for each different level of disturbance/resource. Moreover, areas where the tree mortality driven by the blowdown ranged between 20–50% are those which provide gap space and light conditions, thus favoring a wider range of species with different requirements.

## Discussion

### Disturbance patterns

Mortality rate and structural measurements of the undisturbed forest were similar to those reported in other undisturbed forests nearby [52], [67], [77], [85]. Tree mortality levels observed in the disturbed forest were in accordance with patterns described in earlier studies [9], [10], [25] also in Amazon and even higher than in hurricane-damaged forests [30], [45], [47], [86].

Wind-related mortality caused by snapping and uprooting was greatest in more exposed areas, including plateaus and the top of slopes (Table 2). As a consequence of lower disturbance intensity, structural patterns were not altered by blowdowns in the valleys (Table 1). This may indicate that strong winds did not reach the valley floors, or that the vegetation in valleys is less affected by wind damage, as has been observed in other tropical and sub-tropical forests affected by cyclones [23], [24], [29], [48], [87]. We also did not observe uprooted canopy palm trees in valleys and just 14 snapped and/or standing dead ones. As there is no evidence that caulescent palm tree species that occur in these valleys are less susceptible to wind damage (see species in item 2.2), we assume that valley floors are indeed better protected from strong winds. Although in these forests vegetation damage was partially controlled by abiotic aspects such as wind characteristics and topography [22], [29], [88], resolution of this question requires more information on individual-species traits, such as anatomic-morphological variations among individuals and species, size distribution and populations range, and individual pre-disturbance conditions (pest-attack, biological interactions, age, etc.).

In the disturbed forest, measures of forest structure, including tree density and basal area, were lower four years following the blowdown event (Table 1). The topographic variation of measures of structure was amplified by the landscape driven differences in mortality. Immediate tree mortality effects on tree density and basal area reduction are directly dependent on the disturbance intensity – i.e. the fraction of trees killed by wind (Table 2). In heavily damaged areas with the highest estimated mortality rates, structural measures were lower than the early-successional stages found in other nearby and smaller disturbed forest patches [89] and anthropogenic disturbances in the same forest type [41], [90]. At these sites, forest recovery will depend on species responses to the light gradient in gaps, which was supported for our results related to the third and fourth questions. Within the disturbed areas, the correlation of percent mortality with structural characteristics also showed that areas less affected by wind damage were similar to the undisturbed forest sub-plots.

The observed higher mortality within plateaus and slopes of the undisturbed forest was also related in nearby unaffected *terra-firme* forest, where under regular disturbance regimes, standing death can be expected to be more frequent in plateaus, and uprooting and snapping more frequent in slopes and in valleys [91]. Thus, competition can be more important in plateaus and exogenous disturbances dominant in slopes and valleys, which will cause differences in the size-density distribution of trees related to topographic position [92]. Despite such findings, our study indicates that for the Central Amazon forests, most notably in plateaus and slopes, tree mortality is also regulated by wind disturbance regimes such as blowdowns of varying size.

Although in the undisturbed forest we only observed small gaps, in the disturbed forest large gaps (2000 m^2^) were common and changed forest structure, particularly in plateaus and slopes. Such results suggest that severe disturbances are required to form large gaps. The higher values of tree density and basal area observed in plateaus may indicate a variation in forest vulnerability due differences in species composition, or possibly higher resilience of plateaus.

### Selective mortality patterns

We expected to see large emergent to be more prone to wind damage than smaller ones. While we did observe differences in DBH distribution of dead trees between undisturbed and disturbed forests that suggests lower proportional mortality for smaller trees (despite potential biases introduced because smaller dead trees decompose faster), we also observed no correlation between the DBH of dead trees and sub-plot mortality, indicating that size selective mortality did not depend on disturbance intensity.

Observed reductions in mean DBH and wood density in the live trees in highly damaged areas of the disturbed forest (Figure 2c) likely reflect fast recruitment of light-demanding and fast-growing species with shorter life span and generally lower wood density values [15], [34], [71]. Chambers *et al*. (2009b) compared non-disturbed and smaller blowdown gaps with ∼10 years-succession and reported similar patterns, except for tree density, which was lower in our four-years disturbed plots. The substantial increase in the fraction of small trees with increasing sub-plot mortality also evidentiate the recruitment of shade-tolerant or understory species that usually do not reach the canopy, and thus demonstrates a selective mortality feedback on floristic composition.

Tree diversity in Central Amazon forests is high, and our data agree with those of other studies conducted in the same [52], [67], [93] and in adjacent areas [55], [57], [94], [95]. We observed clear effects of the blowdown mortality on community demographic patterns from low to high disturbance (Figure 3). Part of the observed changes can be attributed to the fast recruitment of species with different syndromes and growth (lateral expansion) in the available gap space, such as *Cecropia sciadophylla* Mart., *Pourouma tomentosa* Mart. ex Miq., *Inga* cf. *paraensis*, *Iryanthera juruensis* Warb., *Protium hebetatum* D. C. Daly, *Mabea speciosa* Müll.Arg. and *Zygia racemosa* (Ducke) Barneby & J. W. Grimes. These species improved their importance within the high disturbance sub-plots by increasing their relative density and frequency. Such patterns may indicate success of establishment and expansion of these populations [81], which in the longer-term may increase species richness and forest diversity [89]. Common tree canopy genera in adjacent areas (e.g. *Couratari*, *Eschweilera*, *Pouteria*, *Licania*, *Micropholis*, *Ocotea*, *Sloanea*, *Swartzia* and *Vantanea*) [67], [90], [93], also presented high importance within the sub-plots with high disturbance (i.e. high mortality). Survivorship of shade-tolerant and slower-growing species from these genera may be attributable to the relatively high wood density (∼0.79 g/cm^3^) [71], small crown footprint, and presence of buttress and supporting roots [54], [56]. Blowdowns are a major disturbance in these forests, thus the greater likelihood of survival and resprouting ability may be reasons for these genera to be of such high importance in the *terra firme* forests of this region [54]–[56], [96].

Blowdown mortality, which directly affected species richness by reducing tree density at a local scale, resulted in the negative correlations between species richness and Shannon diversity with sub-plot mortality (Figure 4a and b). Nevertheless, the rarefaction curve indicate that a slightly higher number of species would be observed for any smaller subsample of individuals taken from low to intermediate disturbance areas, under the assumption of random mixing of individuals (Figure 4c). Interestingly, despite differences in species richness driven by disturbance intensity, the higher number of rare species at low and intermediate disturbance levels indicates that a short-term feedback of blowdown mortality is to promote species richness at the landscape scale, with apparent lower impacts on valleys.

### Disturbance gradient effects and vegetation responses

So far we have focused on the importance of wind disturbance for providing niche space for light-demanding species requiring large canopy gaps for regeneration as a mechanism for promoting species coexistence and thus diversity at the landscape scale. Concomitantly, our data and tests related to the third and fourth questions support that the studied gaps also provide adequate niches for species with different requirements.

Disturbance and selective mortality patterns promoted immediate effects on forest structure and species composition, as evidenced by changes in tree size distribution (Figure 2), species importance between levels of disturbance (Figure 3) and species diversity along the mortality gradient (Figure 4). Nonetheless, blowdown gaps seem to produce long-term effects at the community level. The observed landscape mortality gradient also changed diversity patterns by promoting compositional changes with respect to the fraction of resprouters (Figure 5) and with light-demanding/fast-growing species (Figure 6).

Thus, a second potentially important niche axis is the ability to survive wind disturbance, which may provide a selective advantage to species that are less susceptible to wind damage either because they possess specific morphological and anatomical adaptations lending stability or because they are of small stature and are typically not exposed to wind. Both characteristics may represent a competitive disadvantage in the absence of wind disturbances. In the first case, stability conferred by e.g. high wood density is associated with high construction costs and reduced growth rates – a typical growth-defense tradeoff reported in the literature [8], [71], [97]. In the second case, staying short or retaining a low height/diameter ratio is certainly a disadvantage in the race for light [62].

The positive correlations between disturbance intensity measures and density of resprouters in the disturbed forest (Figure 5) confirms the importance of this regeneration mechanism in gaps created by wind disturbances [31], [47], [48], [86], [98], [99]. The similarities in mean wood density between resprouters and non-resprouters (usually undamaged trees) indicate that resprouting is a regeneration pathway adopted by species with different traits and light requirements, predominantly by smaller trees (DBH≤20 cm). A similar pattern was found in a Caribbean hurricane-damaged forest [45] and indicates that, although canopy trees from higher wood density species may have higher wind resistance, resprouting is not an exclusive regeneration pathway for shade-tolerant, slower-growing or climax species. As for sub-tropical hurricane-damaged forests [46], we hypothesize that in *terra firme* forests of Central Amazon, both early secondary and understory species may benefit from wind disturbances. Finally, the decrease in the mean aggregated wood density in disturbed sub-plots is a direct consequence of the higher fraction of pioneer species which are characterized by low wood densities (Figure 6). Wood density variation among sub-plots was partially explained by sub-plot mortality, which is significant when low wood density pioneers are observed (Figure 6a and b). This greater importance of pioneers in heavily damaged areas corroborates demographic patterns found by assessing species IVI (Figure 3b) and patterns in species composition, which was indicated by the NMDS-test and regressions (Figure 7).

Our data show that species turnover in the 20 most important genera (as quantified by IVI in Figure 3) is due to the increasing importance of both non-pioneer and pioneer species. Indirect evidence for this mechanism is provided by fact that the floristic dissimilarity between undisturbed and disturbed sub-plots seems to be driven by the admixture of light-demanding species (Figure 7a and Figure 8). There was a significant correlation between sub-plot mortality and the position of sub-plots along the first NMDS axis, the latter explaining significant part of the total variation in species composition (Figure 7b). This relationship was higher when considering neighboring-mortality and gap size values (Table 3). However, even in the plots with highest mortality rates (40–70%) there was no complete takeover by classical pioneer local genera (e.g. *Cecropia*, *Conceveiba*, *Croton*, *Goupia*, *Inga*, *Laetia*, *Miconia*, *Pourouma*, *Tachigali* and *Vismia*) or shade-tolerant common genera in undisturbed forests (e.g. *Couratari*, *Eschweilera*, *Licania*, *Pouteria*, *Scleronema*, *Sloanea* and *Swartzia*). These last, maintained up a large fraction of the IVI as indicated by their high proportion in the largest diameter class. Some of the fast growth species we have recorded, especially from the genus *Cecropia*, *Inga*, *Pourouma* and *Tachigali* were also reported as important species during early succession stages of secondary *terra-firme* forests around Manaus [40], [90], [100] and in the upper Rio Negro [41]. In anthropic secondary forests, under non-intensive use and without fire regimes, areas dominated by species from the genus *Cecropia* and *Pourouma* present relatively higher species richness, which hence more rapid plant succession [40], [100].

The variations of genera importance in respect to mortality intensity support classical studies [2], [5], [6], [34] that attempt to classify species into a simplified conception of pioneer species in gaps versus intermediate or late-succession species under undisturbed forest patches. These classical opposing strategies were observed within the 25 most important genera of the disturbed forest (Figure 8), as characterized as high (light-demanding and faster-growing - red lines) and low disturbance specialists (shade-tolerant and slower-growing - green lines). Additionally, our data highlighted alternative successional trajectories related to survival (resistant or unaffected - black lines), resprouting and fast-recruitment (favored under intermediate and high disturbance - blue and orange, respectively).

These alternative successional trajectories have not been observed as important regeneration mechanisms in smaller treefall events. In larger gaps (>2000 m^2^), the canopy emergence of specialized guilds with an optimum at specific levels of disturbance, indicate a smooth occupation of the entire gap space and light conditions. The similar pattern within genera belonging to the same guilds may indicate similar regeneration strategies and historical life. A possible explanation for the success of species from these different genera may be the higher plasticity of traits and alternative resilience mechanisms (such as survival, resprouting and recruitment). As blowdowns are common in these forests [10], [12], [14], these results show that a simplified classification system is not enough to describe successional trajectories of large gaps.

Surprisingly, all the 25 most important genera in the disturbed forest (Figure 3) also figured within the 121 most abundant genera of the main portion of the Amazon [96]. *Protium*, *Pouteria* and *Eperua* characterized the low disturbance specialist guild in the disturbed forest (Figure 8) and took the second, third and seventh position in the Amazonian genera abundance ranking, respectively [96]. *Ocotea*, *Sloanea* and *Couratari* characterized the resistant or unaffected guild in the disturbed forest and took the 14°, 22° and 106° position in the Amazonian ranking, respectively. *Eschweilera*, *Licania*, *Lecythis* and *Swartzia* characterized the intermediate disturbance intensity at the disturbed forest and took the first, fourth, 10° and 17° position in the Amazonian ranking, respectively. *Inga*, *Pourouma* and *Cecropia* characterized the high disturbance specialist guild in the disturbed forest and figured at the sixth, 18° and 30° position in the Amazonian ranking.

The co-occurrence of species with a broad range of life history strategies at intermediate disturbance levels (evidentiated as higher confluence of lines - Figure 8) shows that this blowdown promoted species richness probably by amplifying niches and/or resources. We assume that large gaps (>2000 m^2^) produced by blowdowns can be partly associated to increased diversity and the contrasting dominance of some species in these forests [54], [55], [57], [67], [94], [96]. Variations in genera importance and species composition along the disturbance gradient indicate that gap recovery in large gaps is influenced by mortality patters, most pronounced in plateaus and slopes. Additionally, selective mortality and fast-recruitment highlight genera specialization along the mortality gradient, and indicate that large gaps contain environmental variability that together with species responses and resistance, allow high tree species diversity. In this study, intermediate-disturbance levels had higher species richness suggesting that there was a selective mortality and resulting species turnover in response to the disturbance gradient. This pattern provides evidence that blowdowns allow species richness and diversity through an interaction of wind-damage with species-resistance and resilience. Although classical successional guilds may dominate small treefall gaps, the observed pattern indicate that in large gaps (>2000 m^2^) there are specialists for a wider range of disturbance and light. Thus, we assume that a diverse set of species differing widely in light requirements and recruitment strategies forms the pioneer cohort, thus lending a high resilience towards wind disturbances at the community level.

Our results add new and complementary information about succession and turnover of Western and Central Amazon forests and reinforce that recovery processes for large gaps differ from the trajectory observed in gaps formed by smaller treefall events [2], [6], [15], [31], [86] and secondary forests from anthropic activities, where fire and logging may increase biomass losses [41], [90] and limit species regeneration [40], [100]. Moreover, we revealed a gradient in demographic responses along the entire gradient of disturbance levels. Mortality intensity and gap size seem to influence community composition by filtering shade-tolerant survivors with resprouting ability and favoring more light-demanding and fast-growing species. As already observed in tropical and subtropical regions [26], [29], [32], [47], [98], [101] wind damage depends on species composition and successional stage, which suggest that secondary forests in the Amazon might be more vulnerable and less resilient to windstorms than forests in more advanced successional stage. Thus, for better understanding the blowdown effects on species distribution, forest vulnerability, ecosystem functioning, and the ecological importance of large gaps on species maintenance it is necessary that future studies include dynamic and long-term succession data. In addition, the proliferation of lianas and pioneer tree species in severely disturbed areas may represent an important impediment to seedling establishment [102]. Previous studies in this region showed that it takes an average of ∼18 years for dead trees (>10 cm DBB) to completely decay, although some can take considerably longer [103], so that coarse woody debris and greater surface litter accumulation can have a persistent effect on forest regeneration by covering the soil and acting as a physical barrier, which can favor or exclude species [104], [105]. The persistence of decomposing debris may also alter nutrient and even perhaps water availability to seedlings and regenerating species, all of which will serve as filters that help determine which species recolonize large gaps.

## Conclusion

Blowdown gaps larger than 2000 m^2^ initiate secondary succession providing niches to establish a canopy position for species with a broad range of life history strategies and requirements. Smooth gradients in demographic responses and the existence of specialists for different levels of disturbance can perhaps help explain carbon cycle, maintenance of biodiversity and the recently reported hyperdominance of some tree and palm species in these forests [96]. If forest structure and species composition depend on the intensity and frequency of large disturbance regimes, the intensification of more extreme climate events such as convective storms, may alter forest vulnerability and resilience depending on the successional trajectories. Considering size, vegetation heterogeneity and local logistical limitations, permanent forest monitoring in Amazon must combine remote sensing methods allowing the inclusion of large natural disturbances.
