# Correction: Large-Scale Wind Disturbances Promote Tree Diversity in a Central Amazon Forest

**DOI:** 10.1371/journal.pone.0114769

**Published:** 2014-11-25

**Authors:** The PLOS ONE Staff

There are errors in the published paper. “Section 2.4.1” should be labelled as subsection “Disturbance patterns” and “item 2.2” should be labelled as subsection “Forest inventory.”
